# 

**Published:** 1895-11

**Authors:** 


### TABLE OF CONTENTS.

ORIGINAL COMMUNICATIONS. PAGE

A Contribution to the Study of the Structure of the Dentine,—the so-called "Sheaths of Neumann." By R. R. ANDREWS, A.M., D.D.S., F.R.M.S. . . . . . . . . . . . . . . . . . . . . . . . . . . . 655

What has been done by the Profession in France on Pyorrhœa Alveolaris. By C. N. PEIRCE, D.D.S., Philadelphia . . . . . . . . . . . . . . . . . 660

Judgment an Important Factor in the Preservation of Teeth. By FREDERICK BRADLEY, D.M.D., Newport, R. I. . . . . . . . . . . . . 664

Cause and Being of Teeth. By HENRY BURCHARD, M.D., D.D.S., Philadelphia . . . . . . . . . . . . . . . . . . . . . . . . . . . . 670

REPORTS OF SOCIETY MEETINGS.

American Dental Association . . . . . . . . . . . . . . . . . . . . . 679

American Academy of Dental Science . . . . . . . . . . . . . . . . . 690

Odontological Society of Pennsylvania . . . . . . . . . . . . . . . . 708

National Association of Dental Faculties . . . . . . . . . . . . . . . 709

EDITORIAL.

The National Association of Dental Faculties . . . . . . . . . . . . . 713

OBITUARY.

Thomas H. Chandler, A.B., D.M.D. . . . . . . . . . . . . . . . . . . 718

